# The influence of mitonuclear genetic variation on personality in seed beetles

**DOI:** 10.1098/rspb.2014.1039

**Published:** 2014-12-07

**Authors:** Hanne Løvlie, Elina Immonen, Emil Gustavsson, Erem Kazancioğlu, Göran Arnqvist

**Affiliations:** Animal Ecology, Department of Ecology and Genetics, Uppsala University, Norbyvägen 18D, 75236 Uppsala, Sweden

**Keywords:** behavioural syndromes, *Callosobruchus maculatus*, epistasis, mtDNA, thanatosis, tonic immobility

## Abstract

There is a growing awareness of the influence of mitochondrial genetic variation on life-history phenotypes, particularly via epistatic interactions with nuclear genes. Owing to their direct effect on traits such as metabolic and growth rates, mitonuclear interactions may also affect variation in behavioural types or personalities (i.e. behavioural variation that is consistent within individuals, but differs among individuals). However, this possibility is largely unexplored. We used mitonuclear introgression lines, where three mitochondrial genomes were introgressed into three nuclear genetic backgrounds, to disentangle genetic effects on behavioural variation in a seed beetle. We found within-individual consistency in a suite of activity-related behaviours, providing evidence for variation in personality. Composite measures of overall activity of individuals in behavioural assays were influenced by both nuclear genetic variation and by the interaction between nuclear and mitochondrial genomes. More importantly, the degree of expression of behavioural and life-history phenotypes was correlated and mitonuclear genetic variation affected expression of these concerted phenotypes. These results show that mitonuclear genetic variation affects both behavioural and life-history traits, and they provide novel insights into the maintenance of genetic variation in behaviour and personality.

## 1. Introduction

A fundamental challenge in biology is to understand phenotypic and genetic variation, and the links between them [1]. A large body of research has recently focused on phenotypes where individuals show inter-individual differences and intra-individual consistency in behaviours. Termed ‘animal personality’ [2–4], within-individual consistency in behaviour has now been demonstrated in a broad range of species, ranging from insects to primates [3–5]. Observations of animal personality question our traditional view of adaptive and fully flexible behavioural responses of individuals. However, observations of within-individual consistency of behavioural responses offer explanations for behaviours that seem to be strikingly non-adaptive in an isolated context. Nevertheless, and despite the current attention, our knowledge of genetic variation underlying phenotypic variation in personality is still very limited, and our understanding of the evolution of personality is therefore limited [5,6]. In theory, personalities can result from correlations among behaviours and less plastic traits, such as life-history traits that reduce behavioural plasticity [2,6–8]. Moreover, through negative frequency-dependent selection [9], personality types can be regarded as alternative strategies, in turn explaining maintenance of variation in personality [2,9].

The pace-of-life syndrome (POLS) [10,11] is an integrative framework that aims to understand variation in personality, through covariation between life-history traits and behaviour. The framework is based on growth–mortality trade-offs [7] and has similarities to the concept of a fast–slow life-history continuum (e.g. [12]). The framework incorporates life history, behaviour and also physiological traits [11], and suggests that individuals adopt ‘fast’ or ‘slow’ lifestyles: individuals with slow lifestyles are expected to be less active, more risk-averse and to have a lower metabolic rate and reproductive rate compared with ‘faster’ individuals [10], by analogy with life-history variation across species [13]. Incorporating variation in metabolic phenotypes offers a potential combining factor to the components included in the POLS. This is because metabolic rate is fundamental to energy production and expenditure, thus forming an important potential link between variation in behavioural and life-history traits [14]. Individual variation and consistency in metabolic rates is generally high among individuals [15] (but see [16]). As a consequence, a link between metabolic rate, behaviour and/or life-history traits can offer an explanation for both inter-individual differences and intra-individual consistency in behaviour [7,11].

So far, empirical studies investigating POLS are somewhat inconclusive. A few studies have demonstrated a positive link between personality and metabolic rate [17–19], and between metabolic rate, life-history traits and personality [10,20]. By contrast, other studies have failed to find such a relationship [21–24], or have found a negative relationship between personality, life-history traits and metabolic rates [10,25]. This may, at least in part, be due to taxa-specific effects and/or differences across studies in the methodology used. In addition, the genetic architecture of metabolic rate is not well understood [26–28] and the maintenance of variation in POLS thus remains unclear. Those studies that have demonstrated a relationship between personality, metabolic rate and life-history traits suggest that genes with major pleiotropic effects may be involved [3,20].

The level of additive genetic variance in metabolic rate is generally low among both vertebrates [29–33] and invertebrates [34–36], although there are clearly some exceptions to this [37,38]. Considering the fact that repeatabilities of metabolic rate tend to be high [16], this suggests that the genetic architecture of metabolic rate may generally be complex [34,39,40]. Arnqvist *et al*. [28] recently suggested that mitonuclear epistasis may be an underestimated source of genetic variation in metabolic phenotypes. Because products of the mitochondrial and nuclear genomes jointly form the oxidative phosphorylation pathway, the major generator of ATP in the cell, it is easy to envision how epistatic interactions between these two genomes could interact [41]. Our view of mitochondrial genetic variation is currently changing [42–45], and it is now widely recognized that mitonuclear genetic variation often affects important life-history traits and fitness (e.g. [28,43,45–50]). The fact that mitonuclear genetic variation can affect life-history traits such as metabolism [28] and longevity [50] suggests that it may also affect POLS and personality, although this possibility remains unexplored. Empirically, investigation of the role of mitonuclear genotypes on personality may be hampered by the logistics of disentangling the relative contributions of nuclear versus mitochondrial genes. However, this can be achieved in amenable model systems by constructing mitonuclear introgression lines where mitochondrial (i.e. cytoplasmic) genomes are introgressed into controlled nuclear genetic backgrounds (e.g. [28,46,49,51]).

The use of insect models is increasing in personality research, and the personality gradients described show large similarities with those documented in vertebrates. For example, Chinese bruchid beetles (*Callosobruchus chinensis*) differ in activity [52], firebugs (*Pyrrhocoris apterus*) vary along a shy–bold continuum in exploration and activity [53], mustard leaf beetles (*Phaedon cocheariae*) vary in their duration of death-feigning and activity [54], confused flour beetles (*Tribolium confusum*) vary in death-feigning [55], field crickets (*Gryllus integer*) vary in boldness [56] and clonal pea aphids (*Acyrthosiphon pisum*) vary in their predator responses [57]. Links between behaviour, life history and physiology have been documented in insect species that have flight-reproduction or migration syndromes [58]. Niemelä *et al*. [56,59] demonstrated a ‘live fast, die young’ syndrome in field crickets, although patterns between life-history traits and personality were not fully consistent with POLS (e.g. the association between boldness and body size was negative, and not positive as predicted by POLS [59]). This shows that the relationships among traits included in POLS are still not fully understood. Further investigation of the links between life-history traits and behaviour, including their underlying mechanism, are therefore needed to improve our understanding of these relationships.

Here, we explore covariation between life-history traits and behavioural traits, and assess whether such covariation is affected by mitonuclear genotypes. As a model system, we employ the seed beetle *Callosobruchus maculatus*. In this system, previous work has demonstrated genetic covariance between suits of life-history traits, including lifespan, body size and metabolic rate (e.g. [60,61]). Further, mitonuclear genotype is known to affect life-history traits such as metabolic rate [28] and growth rate [51], and recent work has unveiled selection on mitonuclear genotypes [46]. Using mitonuclear introgression lines, we demonstrate that mitonuclear interactions indeed affect personality, and that behaviour and life-history traits are correlated across genotypes, as predicted by POLS.

## 2. Material and methods

### (a) Study subject

In *C. maculatus*, mated females cement their eggs to the surface of host beans and larvae borrow into the bean at hatching [62]. Adult beetles emerge from the host and are facultative aphagic (i.e. they obtain all resources necessary for successful survival and reproduction during their larval stages [62]). Seed beetles are pests of stored legumes, and the laboratory environment thus represents a close approximation of such ‘natural’ conditions (e.g. [63]).

### (b) Stock populations and introgression lines

Outbreed stocks of three populations originating from Brazil, California (USA) and Yemen were used to generate nine types of mitonuclear introgression lines fixed for fully crossed combinations of distinct mitochondrial and nuclear lineages, each line replicated three times (i.e. new 27 lines were generated in total). Repeated backcrossing (15 generations) of mitochondrial genes into a specific nuclear background disassociates each of the sampled mitochondrial genomes from the nuclear genome it was originally associated with, replacing it with a novel nuclear genome. As a result, each of the three mitochondrial genomes are expressed in three distinct but genetically variable nuclear genetic backgrounds. We used beetles from the 16–19th generation of backcrossing procedure described by Kazancioglu & Arnqvist [46]. The three stock populations used were selected from a larger set of potential populations, as previous research has shown that their mtDNA genotypes differ and that mitonuclear genetic variation across these populations has phenotypic effects [28,51].

When creating mitonuclear introgression lines, the entire cytoplasm is introgressed into different nuclear genetic backgrounds. Many insects are infected by maternally inherited, cytoplasmic bacteria, and this can potentially confound the results of experiments that aim to investigate mitochondrial genetic effects. Here, we term and interpret cytoplasmic effects observed as being mitochondrial in origin based on three facts. First, infections with endosymbiontic bacteria (e.g. *Wolbachia*) have been carefully screened for in many *C. maculatus* populations, including those used here, but have never been detected [64]. Second, to preclude the possibility that our introgression lines may nevertheless have harboured cytoplasmic bacterial infections, we treated all introgression lines with an effective antibiotic treatment between generations 13 and 14 [46]. Third, mtDNA sequence divergence across these haplotypes is quantitatively associated with phenotypic divergence [28].

Beetles were reared on black-eyed beans (*Vigna unguiculata*), at 50% RH, 29°C and at a 12 L : 12 D cycle. Each population was kept in a single glass jar (approx. 30 × 10 cm, height × diameter). To generate beetles used in this study, single beans with eggs of known age were collected from the population before adult beetles emerged and kept in 1.5 ml Eppendorf tubes provided with an air hole in the lid. No food or water was provided for the beetles, unless otherwise stated (see ‘Mating and lifespan’ below). The experiment was conducted in four successive blocks that were separated in time (i.e. conducted on four subsequent generations).

### (c) Life-history traits

We collected data on two different life-history traits on experimental beetles: (i) emergence weight (weight within 12 h of adult emergence) and (ii) lifespan (in days, determined by spot checks twice per day). Weight was measured to the nearest 0.00001 g (Sartorius Genius ME 235P), all individuals were weighed at least two times, and the mean value was used for further analyses.

### (d) Behavioural assays

To investigate variation in behavioural responses, single beetles (*n* = 932) were observed in a novel arena test for 10 min in the afternoon (13.00–18.00 local time) of the day after they had emerged (i.e. when 1 day old). The novel arena consisted of a round 15 cm Petri dish divided into nine sub-areas (one centre and eight equal-sized peripheral areas). This type of test was originally developed to determine activity and exploration propensity (see references in [54]), and similar circular arenas have successfully been used to measure variation in exploration and activity in insects (e.g. [53,54]).

Assays were initiated by inducing beetles into tonic immobility (also called death-feigning behaviour or thanatosis [65,66]) by rapidly shaking the Petri dish three times horizontally towards the observer's hand, thereafter recording ‘latency to walking’ (in seconds) as a measure of when the beetle came out of tonic immobility. Tonic immobility is an anti-predator behaviour observed in various animal taxa including insects (see references in [52,55,67]). Other recent studies have used similar approaches to manually induce tonic immobility in other insects [52–55,65,67] and have used latency to walking to score variation in insect personality (e.g. [52–55,59,65,67]).

To score variation in activity, we recorded whether the beetle was ‘still’ (i.e. no movement, post-thanatosis), ‘preening’ (i.e. preening antennae, legs, etc.) or ‘walking’ (i.e. the beetle was walking or running) once every 30 s for the 10 min the individual was observed in the novel arena assay. The proportions of time a beetle conducted each of these behaviours were calculated by dividing the number of occasions the beetle was recorded to be, for example, ‘still’, over the total number of times it was recorded as being either ‘still’, ‘preening’ or ‘walking’ (i.e. having left the tonic immobility state). These variables were called ‘proportion still’, ‘proportion preening’ and ‘proportion walking’, respectively, capturing variation in the overall activity level of the beetles. Further, ‘activity’, capturing variation in speed and walking distance, was calculated by dividing the number of sub-area transitions the beetle made between the nine sub-areas in the novel arena by the time that each beetle spent not being in a tonic immobility state.

### (e) Consistency of behavioural responses

We repeated the novel arena assay for 85 beetles, chosen to represent both sexes and a variety of lines, during the afternoon the day after their initial assay, enabling us to investigate the temporal consistency of their behaviour.

In addition, 44 different beetles (representing both sexes and a variety of lines) were tested in an additional behavioural assay to investigate the consistency of behavioural responses across contexts, in a test designed to capture functionally similar behavioural responses. These beetles were first (during the morning of the day after they had emerged) exposed to a vertical test that was performed using an opaque tube (10 cm long, 1.5 cm diameter). Each beetle was first placed on a flat surface and the tube was placed over the beetle. ‘Latency to walking’ was here recorded as the latency (in seconds) until the beetle started climbing up the tube, and ‘activity’ was estimated by recording the time (in seconds) it took for the beetle to climb to the top of the tube. The maximum time was set to 5 min. After the vertical test, beetles were observed in the novel arena assay (see above). Latency to come out of tonic immobility was also investigated in an additional, horizontal test in the afternoon on day 2 of adult life. In this test, the beetle was dropped from 5 cm of height, which typically induced tonic immobility, and ‘latency to walking’ was then measured by the latency until they started moving (in second). Maximum time for this test was set to 5 min.

### (f) Mating and lifespan

A sample of 451 of the beetles was mated at day 2 of adult age (i.e. subsequent to behavioural assays) to a partner selected randomly from the same line and emergence date. These beetles were also provided with sugar solution (ad libitum) for 30 min on day 10. Analyses of variation in lifespan including these beetles enabled us to investigate the collective effect of mating and feeding status on lifespan.

### (g) Statistical analyses

#### (i) Genotyping of introgression lines

To validate the integrity of the lines, all propagating females from the 27 introgression lines were sequenced for a single polymorphic mtDNA marker at generation 16 (for details on genotyping, see [46]). This effort yielded a few females with ambiguous sequence data from one line, which was therefore not used further. Thus, a total of 26 lines were analysed. The genotyping also showed that one of the three replicate Californian lines carried a haplotype slightly different to the other two. Consequently, we coded mtDNA haplotypes as belonging to any of four haplotypes in the analyses reported here (Brazil, California 1, California 2 and Yemen).

#### (ii) Consistency in behaviour

The repeatability of behavioural responses of individual beetles observed twice in the novel arena test was calculated from a one-way ANOVA according to Nakagawa & Schielzeth [68]. For beetles observed in different behavioural tests, estimates of stability in functionally similar behaviours were obtained from Spearman's rank-order correlations of behaviours across tests. Analyses were carried out in SAS v. 9.3.

#### (iii) Influence of mitonuclear interactions

*Principle component analysis*. A principle component analysis based on the correlation matrix was conducted in GenStat v. 10.2 to describe variation in overall activity level of beetles, including the behavioural responses ‘proportion still’, ‘proportion preening’ and ‘proportion walking’. The first PC (*λ* = 1.94, explaining 64% of the variation) primarily loaded with ‘proportion walking’ (−0.99) and ‘proportion resting’ (0.81), thus describing how inactive beetles were, was termed ‘PC1 activity’. The second component (*λ* = 1.06, explaining 35% of the variation) loaded with ‘proportion preening’ (−0.84), and with ‘proportion resting’ (0.59), primarily described the relative time beetles spent preening versus resting and was here termed ‘PC2 preening’.

*Univariate analyses*. Variation in life-history traits and behavioural responses was investigated through a series of linear mixed models (LMM) in GenStat v. 10.2, with sex (i.e. male, female), nDNA type (i.e. Brazil, California, Yemen) and mtDNA type (i.e. Brazil, California 1, California 2, Yemen) entered as fixed effect factors. The epistatic interaction terms nDNA × mtDNA and nDNA × mtDNA × sex were also included in these models. Whether the beetles were mated or not (i.e. their mating status) was entered as an additional fixed effect for the data on variance in lifespan, together with the interactions between mating status and variation in nDNA and mtDNA types. These interactions were, however, non-significant and were dropped from the final model. Emergence weight was added as a covariate to all models. Line ID (the replicate introgression line the beetle came from, 1–26) and generation (i.e. 1–4) were included as two random-effect factors in these models.

Disruption of coadapted genotypes can lead to functional incompatibilities. This can be observed as a consequence of non-coevolved mitonuclear combinations. To investigate this possibility, we ran LMM models in SAS v. 9.3 for response variables where we had observed a mtDNA × nDNA interaction with whether the mitonuclear combinations were coevolved or not added as a fixed effect. Here, line ID and generation were retained as random-effect factors. In these analyses, the two Californian mtDNA types were not distinguished (as both have potentially coevolved with the Californian nuclear genome).

*Multivariate analyses*. Under POLS, behavioural and life-history traits to some extent share a common genetic architecture. To test this, we first asked whether behavioural traits and life-history traits covary, using a canonical correlation analysis including all behavioural traits as one set and all life-history traits as the other set, where both sets were partitioned by generation and line ID. We then assessed whether this covariation is affected by mitonuclear genotype, by treating the first pair of canonical variates as collective response variables in a multivariate LMM in GenStat v. 10.2. This model included mtDNA, nDNA, sex and their two-way interactions as fixed-effect factors and generation as a random-effect factor.

## 3. Results

### (a) Life-history traits

#### (i) Univariate analyses

The two life-history traits of emergence weight and lifespan were not significantly influenced by the interaction between the genomes (table 1). However, both nuclear background and sex significantly explained variation in both traits (mean ± s.e.: emergence weight, nDNA: Brazil: 439.32 ± 6.28, California: 457.81 ± 6.40, Yemen: 475.85 ± 6.41; sex, female: 538.09 ± 5.03, male: 385.54 ± 3.19; lifespan, nDNA: Brazil: 17.72 ± 0.26, California: 19.87 ± 0.33, Yemen: 19.49 ± 0.39; sex, female: 22.59 ± 0.27, male: 15.89 ± 0.22). Lifespan was also affected by the interaction between nDNA and sex, whether beetles were mated or not (mated: 20.31 ± 0.32, not mated: 18.30 ± 0.24), and by the initial emergence weight of beetles, such that large beetles live longer (table 1).

**Table 1.** Mitonuclear genetic effects on life-history traits in seed beetles. mtDNA, mitochondrial haplotype; nDNA, nuclear genetic background. *p* < 0.05 is highlighted in italic.

variables	*F*	ndf	ddf	*p*
emergence weight				
mtDNA	0.49	3	13.8	0.69
nDNA	7.34	2	13.8	*0.007*
mtDNA × nDNA	1.10	6	13.8	0.41
sex	836.71	1	1033.8	<*0.001*
mtDNA × sex	0.15	3	1033.7	0.93
nDNA × sex	2.47	2	1034.2	0.09
mtDNA × nDNA × sex	0.74	6	1033.5	0.62
lifespan				
mtDNA	0.86	3	13.8	0.49
nDNA	7.60	2	14.1	*0.006*
mtDNA × nDNA	1.94	6	13.9	0.15
sex	400.54	1	976.4	<*0.001*
mtDNA × sex	0.52	3	984.1	0.67
nDNA × sex	4.62	2	981.9	*0.01*
mtDNA × nDNA × sex	1.24	4	980.6	0.28
mated	4.75	1	214.6	*0.03*
emergence weight	113.3	1	727.3	<*0.001*

### (b) Behaviours

#### (i) Consistency of behaviours

Latency to walking and activity, proportion resting and proportion preening were generally not strongly correlated (table 2), thus these variables are describing partly different aspects of individuals' behaviours. Beetles were consistent over time in how active they were (table 3), and were consistent across assays in their latency to walking and activity (table 4).

**Table 2.** Within-assay correlations of behavioural responses of seed beetles in the novel arena test. Spearman's rank correlations (*n* = 85; *r*_s crit_ = 0.216, *α* = 0.05).

	latency to walking	activity	proportion resting	proportion preening
activity	0.04			
proportion resting	0.23	−0.73		
proportion preening	−0.29	−0.57	0.14	
proportion walking	−0.02	0.88	−0.86	−0.60

**Table 3.** Repeatability of behaviour in seed beetles over time in the novel arena test. *p* < 0.05 is highlighted in italic (*n* = 85).

	*R* ± s.e.	*p*
latency to walking	0.16 ± 0.11	0.075
activity	0.25 ± 0.10	*0.011*
proportion resting	−0.11 ± 0.11	0.83
proportion preening	0.02 ± 0.11	0.43
proportion walking	0.27 ± 0.10	*0.005*

**Table 4.** Consistency of behaviour in seed beetles across contexts. DT, Drop Test; VT, Vertical Test; NA, Novel Arena. Spearman's rank correlations (*n* = 44; *r*_s crit_ = 0.305, *α* = 0.05).

	latency to walk DT	latency to walk VT	latency to top VT
latency to walking NA	0.42	0.16	0.27
activity NA	−0.06	−0.40	−0.43

#### (ii) Univariate analyses

**Figure 1.**
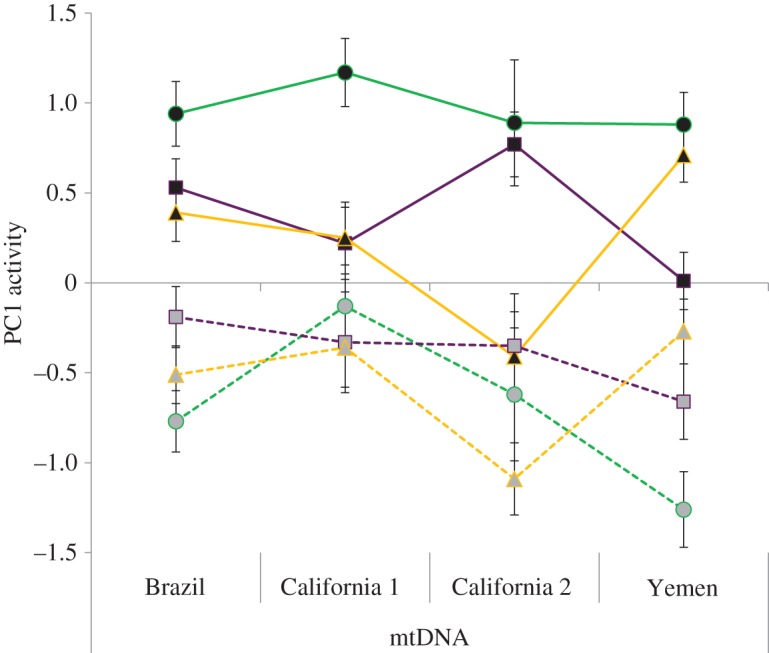
Variation in behaviour of introgressed lines of seed beetles. Activity (‘PC1 activity’, negative values denote higher activity) was affected by sex (females, black symbols, solid lines; males, grey symbols, dotted line), but also by mitonuclear interactions (origin of nuclear genome: Brazil, circles; California, squares; Yemen, triangles; origin of mitochondrial genome, mtDNA: Brazil, California 1, California 2, California haplotype 1 and 2, respectively, Yemen). (Online version in colour.)

Overall activity was influenced by the mtDNA × nDNA interaction (figure 1 and table 5).

**Table 5.** Factors affecting variation in behaviour of seed beetles. mtDNA, mitochondrial haplotype; nDNA, nuclear genetic background (see main text for further details). mtDNA, mitochondrial haplotype; nDNA, nuclear genetic background. *p* < 0.05 is highlighted in italic.

variables	*F*	ndf	ddf	*p*
latency to walking				
mtDNA	1.02	3	13.9	0.41
nDNA	1.86	2	14.1	0.19
mtDNA × nDNA	1.79	6	13.9	0.17
sex	2.30	1	656.3	0.13
mtDNA × sex	1.22	3	848.7	0.30
nDNA × sex	3.27	2	848.6	*0.039*
mtDNA × nDNA × sex	1.75	6	848.3	0.11
emergence weight	2.85	1	273.8	0.09
activity				
mtDNA	1.23	3	14.2	0.34
nDNA	0.24	2	14.6	0.79
mtDNA × nDNA	3.11	6	14.3	*0.037*
sex	130.33	1	858.6	<*0.001*
mtDNA × sex	1.00	3	849.5	0.39
nDNA × sex	7.03	2	850.4	<*0.001*
mtDNA × nDNA × sex	0.16	6	849.4	0.99
emergence weight	0.08	1	852.6	0.78
‘PC1 activity’				
mtDNA	1.67	3	14.2	0.22
nDNA	1.48	2	14.7	0.26
mtDNA × nDNA	5.67	6	14.4	*0.003*
sex	180.41	1	855	<*0.001*
mtDNA × sex	0.88	3	850.3	0.45
nDNA × sex	15.12	2	851.2	<*0.001*
mtDNA × nDNA × sex	0.80	6	850.1	0.57
emergence weight	4.6	1	800.5	*0.032*
‘PC2 preening’				
mtDNA	0.45	3	14.2	0.72
nDNA	7.85	2	14.5	*0.005*
mtDNA × nDNA	0.81	6	14.3	0.58
sex	25.22	1	833.4	<*0.001*
mtDNA × sex	1.93	3	846.9	0.12
nDNA × sex	0.38	2	847.2	0.69
mtDNA × nDNA × sex	0.28	6	846.7	0.94
emergence weight	0.00	1	689	0.95

The observed interactions of the mitochondrial and nuclear genomes were not caused by disruption of mitonuclear combinations in introgressed lines (test of effects of interruption of coevolved mitonuclear combinations or not, latency to walking, *F*_1,876_ = 0.02, *p* = 0.89; activity, *F*_1,876_ = 1.36, *p* = 0.24; proportion resting, *F*_1,876_ = 1.48, *p* = 0.22; proportion preening, *F*_1,875_ = 0.09, *p* = 0.77; proportion walking, *F*_1,876_ = 1.06, *p* = 0.31; PC1 activity, *F*_1,875_ = 1.16, *p* = 0.28; PC2 preening, *F*_1,875_ = 0.70, *p* = 0.40).

Further, males and females differed in all behavioural responses recorded (latency to walking, mean ± s.e., female: 51.49 ± 4.62, male: 42.25 ± 3.80; activity, female: 0.0412 ± 0.00175, male: 0.0707 ± 0.00216; PC1 activity, female: 0.55 ± 0.057, male: −0.501 ± 0.062; and PC2 preening, female: −0.139 ± 0.058, male: 0.165 ± 0.037; table 5). The nuclear genetic background (nDNA) of the beetles had a series of effects on their behaviour, including latency to walking (in interaction with sex, mean ± s.e., females, Brazil: 62.1 ± 9.71, California: 50.03 ± 6.77, Yemen: 42.49 ± 7.32; males, Brazil: 35.31 ± 6.85, California: 58.22 ± 7.58, Yemen: 33.62 ± 5.23), activity (in interaction with sex, mean ± s.e., females, Brazil: 0.031 ± 0.0026, California: 0.047 ± 0.003, Yemen: 0.045 ± 0.0031; males, Brazil: 0.071 ± 0.0039, California: 0.067 ± 0.0033, Yemen: 0.018 ± 0.0062), PC1 activity (in interaction with sex, mean ± s.e., females, Brazil: 0.98 ± 0.10, California: 0.32 ± 0.09, Yemen: 0.37 ± 0.09; males, Brazil: −0.70 ± 0.12, California: −0.37 ± 0.11, Yemen: −0.50 ± 0.10) and PC2 preening (mean ± s.e., Brazil: 0.207 ± 0.064, California: 0.092 ± 0.053, Yemen: −0.027 ± 0.056) (table 5).

### (c) Multivariate analyses

The canonical correlation analysis revealed significant phenotypic covariation between behavioural and life-history traits (first set: *R* = 0.35, *χ*^2^ = 110.8, d.f. = 8, *p* < 0.001; second set: *R* = 0.03, *χ*^2^ = 0.84, d.f. = 3, *p* = 0.84). The canonical loadings of the first set of canonical variates showed that beetles that were less active (activity = −0.64; PC1 activity = 0.91, latency to walking = 0.18; PC2 preening = −0.33) were also those that were heavier and lived longer (emergence weight = 0.98; lifespan = 0.69).

**Figure 2.**
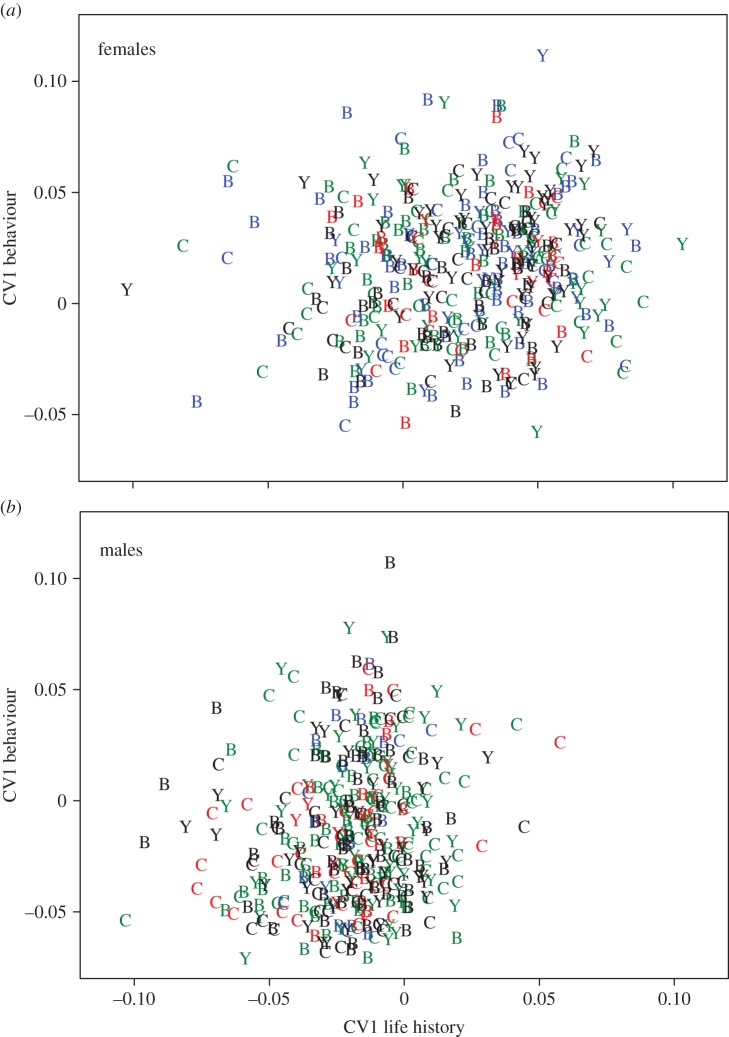
Mitonuclear interactions affect the relationship between behaviour and life-history traits in both female and male seed beetles. CV behaviour (lower value denotes higher activity) and CV life history are the first set of canonical variates, describing covariation between behaviour and life history. Overall, males were more active and showed a shorter lifespan than females (see §2g for further details). Nuclear backgrounds: B, Brazil; C, California; Y, Yemen. (Online version in colour.) Mitochondrial haplotypes: Brazil, green; California 1, blue; California 2, red; Yemen, black.

Further analysis of the pattern of covariation (table 6 and figure 2) showed that the dominant source of covariation was sexual dimorphism. However, the extent of sexual dimorphism was influenced by nuclear genotype. Further, we found mitonuclear genetic effects (table 6), such that the interaction between the mitochondrial and nuclear genome affected the location of beetles along the major axis of covariation in behavioural and life-history traits.

**Table 6.** Factors affecting the covariation between behavioural and life-history traits in seed beetles. In this multivariate LMM, the first set of canonical variates was used as the response variable (see main text for further details). mtDNA, mitochondrial haplotype; nDNA, nuclear genetic background. *p* < 0.05 is highlighted in italic.

variables	Wald statistic	df	*p*
mtDNA	6.92	6	0.33
nDNA	44.75	4	<*0.001*
sex	1023.19	4	<*0.001*
mtDNA × nDNA	26.46	12	*0.009*
mtDNA × sex	3.16	9	0.96
nDNA × sex	28.06	6	<*0.001*

## 4. Discussion

We found that epistatic interactions between mitochondrial and nuclear genes affect the general activity of seed beetles. Variation in activity was consistent within individuals, thus describing personality differences among beetles. In addition, life-history traits and behaviours covaried and mitonuclear interactions affected the joint phenotype described by this covariation. Overall, our results are consistent with the POLS hypothesis and they show that mitochondrial genes may affect variation in pace of life. In this sense, our results contribute to a growing number of studies demonstrating functional effects of variation in mitochondrial genes. Below, we discuss each of these insights.

Studies investigating variation in personality have recently increased in taxonomic width to include also studies of variation in personality in insects. Why individuals show personality has been suggested to be due to constraints in behavioural plasticity and/or because personality types represent alternative strategies with overall equal fitness [2]. The use of insect model systems can potentially improve our understanding of the evolution of personality due to amenability of such systems. Insects have been shown to exhibit similar personality variation as that found in other taxa, describing variation in activity, exploration, boldness and responses to predator attacks (e.g. [52–54,56,57,59]). Here, we demonstrate that individual seed beetles vary in activity and thanatosis, behaviours with similarities to personality gradients describing variation in exploration and boldness in other studies.

Tonic immobility (or thanatosis, death-feigning) is a general phenomenon. Selection for short and long duration, for example work in flour beetles [65,67] and Chinese bruchid beetles [52], has shown that the trait has a genetic component. We investigated tonic immobility of beetles with different mitonuclear combinations and while nuclear factors did significantly affect variation in this behaviour, mitonuclear interactions did not. Recent work shows that the dopamine pathway underlies trait variation in flour beetles [67]. Brain dopamine is a neurotransmitter that regulates behaviour in many insects (see references in [67]) and associations between variation in dopamine receptor genes and personality has been demonstrated in several vertebrates (e.g. great tits *Parus major* [69], humans [70]). Variation in tonic immobility in seed beetles may thus be better explained by variation in the dopamine pathway, rather than by mitonuclear interactions that relate to overall metabolic rate. This possibility warrants further investigation.

By contrast, we found that overall activity was affected by mitonuclear genetic interactions. The observed effects were not due to disruption of coadapted mitonuclear combinations, as would have been expected if disruption had resulted in functional incompatibilities [71]. Instead, we observed that certain evolutionarily novel mitonuclear combinations generated beetles that were more active, compared with beetles from reconstituted lines with the originally coexpressed genomes. Activity was strongly affected by sex, males being more active compared with females, suggesting sex-specific selection on activity in the species. Further, variation in activity was linked to variation in life-history traits, and relatively more active beetles were lighter and lived for a shorter time. This axis of covariation is well aligned with that predicted by the POLS hypothesis. Importantly, the location of beetle phenotypes along this axis was affected by a mitonuclear interaction. This result shows that the two genomes interact in affecting variation in life history and behaviour, which in turn are linked and describe a concerted syndrome. While flight-reproduction syndromes and migration syndromes have previously been described in insects [58], a POLS has to our knowledge only been explicitly described in field crickets [56,59]. Covariation between behaviour morphology and life-history traits consistent with a POLS has however been documented in Chinese bruchid beetles [52,72]. Our demonstration of a link between mitonuclear genotype and behaviour and life-history traits suggest that genetic variation in metabolic parameters may in part be an underlying causal factor of the POLS. Metabolic rate is a candidate for a central and deeply rooted trait with a range of cascading effects on life-history traits and behaviour [15,73]. Previous work in our study species has shown that mitochondrial genes can explain variation in metabolic phenotypes [28,51]. Interestingly, the fact that we found strong sexual dimorphism in location along the ‘POLS axis’ is consistent with the view that males are under selection to ‘live fast and die young’ in many taxa [74], including seed beetles [75], whereas females are not. It is therefore possible that sexually antagonistic selection [76] on a POLS may contribute to the maintenance of genetic variation in metabolism, and thus also behavioural and life-history phenotypes. Trade-offs that would emerge from such a scenario could underlie variation in personality and explain variation in behavioural responses of individuals [7,8]. The observed mitonuclear effects on behaviour as well as mitonuclear effects on concerted behavioural/life-history phenotypes also suggest that these effects may be mediated through variation in metabolic rate. Arnqvist *et al*. [28] demonstrated that mitonuclear interactions affected variation in metabolic rate in *C. maculatus* and suggested that variation in mitochondrial genes may play a more important role in life-history evolution than is generally appreciated. Here, we expand this suggestion to also include personality, adding to the growing number of studies challenging the traditional view that standing genetic variation in mtDNA is neutral (e.g. [45–47]). Although the genetic architecture of metabolism is complex [28], our work should encourage efforts along this line to improve our understanding of the underlying physiological mechanism of covariation between behaviours and life-history traits. Ballard & Melvin [41] suggested that investigations of the bioenergetics of mitochondrial genetic variation may further our understanding of the proximate link between mtDNA and behavioural/life-history phenotypes. This could, for example, include investigation of variation in ATP and reactive oxygen species (ROS) production, which may directly and indirectly affect a range of phenotypic traits [41,77,78].

The general problem of understanding the maintenance of non-neutral genetic variation [79] is exacerbated for mtDNA, because the mitochondrial genome shows maternal inheritance, has a relatively low effective population size, is haploid and generally does not recombine. Although epistasis in general can promote the maintenance of genetic variation [80], theory suggests that mitonuclear interactions show only limited ability to act to maintain variation in mtDNA [47]. We note here that, if mitochondrial haplotypes generally affect behavioural and life-history syndromes, the negative frequency-dependent selection that is thought to contribute to the maintenance of variation in personality (e.g. [9]) may also promote the maintenance of genetic variation in mtDNA within populations. In light of this possibility, it is interesting to note that Kazancioglu & Arnqvist [46] recently documented negative frequency-dependent selection on mtDNA haplotypes in laboratory populations of *C. maculatus*.

## 5. Conclusion

By using experimentally constructed mitonuclear introgression lines, we show that mitonuclear interactions affect phenotypes along an axis of covariation between life-history traits and behaviour in seed beetles. Overall activity of individuals was consistent both within and across contexts, thus describing variation in personality. Our results therefore show that mitonuclear genetic effects affect phenotypic variation in personality. Further, our results are consistent with a sexually dimorphic POLS, where more active beetles are smaller and show reduced lifespan, and males being more active than females. This suggests that sexually antagonistic selection on POLS and negative frequency-dependent selection on POLS phenotypes may both contribute to the maintenance of genetic variation in personality. We suggest that future work should explore the link between POLS phenotypes, metabolic rate and fitness, and should consider the possibility that genetic variation in mitochondrial genes may affect the evolution of personality.

## Data accessibility

Data are deposited in the Dryad repository (doi:10.5061/dryad.qk27t).
